# Temperature- and pH-Sensitive Nanohydrogels of Poly(*N*-Isopropylacrylamide) for Food Packaging Applications: Modelling the Swelling-Collapse Behaviour

**DOI:** 10.1371/journal.pone.0087190

**Published:** 2014-02-10

**Authors:** Clara Fuciños, Pablo Fuciños, Martín Míguez, Issa Katime, Lorenzo M. Pastrana, María L. Rúa

**Affiliations:** 1 Grupo de Bioquímica, Departamento de Química Analítica y Alimentaria, Universidad de Vigo, Ourense, Spain; 2 Grupo de Reciclado y Valorización de Materiales Residuales (REVAL), Instituto de Investigaciones Marinas (CSIC), Vigo, Spain; 3 Grupo de Nuevos Materiales y Espectroscopía Supramolecular, Departamento de Química Física, Universidad del País Vasco, Leioa, Spain; University of Waterloo, Canada

## Abstract

Temperature-sensitive poly(N-isopropylacrylamide) (PNIPA) nanohydrogels were synthesized by nanoemulsion polymerization in water-in-oil systems. Several cross-linking degrees and the incorporation of acrylic acid as comonomer at different concentrations were tested to produce nanohydrogels with a wide range of properties. The physicochemical properties of PNIPA nanohydrogels, and their relationship with the swelling-collapse behaviour, were studied to evaluate the suitability of PNIPA nanoparticles as smart delivery systems (for active packaging). The swelling-collapse transition was analyzed by the change in the optical properties of PNIPA nanohydrogels using ultraviolet-visible spectroscopy. The thermodynamic parameters associated with the nanohydrogels collapse were calculated using a mathematical approach based on the van't Hoff analysis, assuming a two-state equilibrium (swollen to collapsed). A mathematical model is proposed to predict both the thermally induced collapse, and the collapse induced by the simultaneous action of two factors (temperature and pH, or temperature and organic solvent concentration). Finally, van't Hoff analysis was compared with differential scanning calorimetry. The results obtained allow us to solve the problem of determining the molecular weight of the structural repeating unit in cross-linked NIPA polymers, which, as we show, can be estimated from the ratio of the molar heat capacity (obtained from the van't Hoff analysis) to the specific heat capacity (obtained from calorimetric measurements).

## Introduction

Active packaging systems are an innovative tool for food preservation consisting in packages that interact with the food and its environment playing a dynamic role to extend the food shelf life [1]. Antimicrobial active packaging systems release antimicrobial compounds, such as bacteriocins [2], [3], organic acids [4], [5], potassium sorbate [6], [7], or pimaricin (natamycin) [8], [9], that prevent the growth of spoilage microorganisms and food pathogens. Active packaging allows us to reduce the amount of antimicrobial compounds incorporated into the food matrix to grant the desired shelf-life or safety levels. However, in conventional active packaging systems the mass transfer rate is often controlled by the concentration difference between the food and its environment and the packaging materials. As a result, a proper delivery control is not possible and, for a particular system (food matrix + active packaging device + initial antimicrobial load in both packaging and food), the release rate will be a decay function of time. Thus, in these systems, preservatives are mainly released at the beginning of the storage period, which is the least opportune time, since food is still freshly packaged and the microbial load is low.

For these reasons, the new trends in active packaging systems for food applications are placing more emphasis on developing smart delivery systems, in which the release of preservatives could act as a response to environmental triggers (e.g. temperature), and could be modulated so that the amount of preservative released would be proportional to the level of potential spoilage. Nanoparticles of poly (*N*-isopropylacrylamide) (PNIPA) could be used as smart delivery devices to be incorporated to active packaging systems. This material form thermosensitive hydrogels and thus can be used to control the release of food preservatives as a response to temperature changes during the storage. PNIPA has hydrophilic amide groups and hydrophobic isopropyl side chains, so it is soluble in both water at room temperature and low polarity organic solvents. Side chains of PNIPA hydrogels possess an optimal hydrophilic/hydrophobic balance that allows the formation of hydrogen bonds between water molecules and hydrophilic groups of the hydrogel. Below the lower critical solution temperature (LCST), the strong hydrogen bonds established between the hydrophilic groups and water exceeds the unfavourable free energy associated with exposure of hydrophobic groups to water, leading to good polymer solubility. As the temperature increases above the LCST, hydrogen bonds are disrupted and hydrophobic interactions among the hydrophobic groups are dramatically strengthened, which leads to the collapse of the polymer chains and thus the phase transition of the hydrogel network [10], [11].

The collapse pattern can be easily modulated by varying the polymer composition, thus the release behaviour can be also altered, which allows for accurately controlling the delivery of previously loaded active compounds. For instance, the addition of ionic hydrophilic comonomers, such as acrylic, methacrylic or itaconic acid, to the PNIPA polymer leads to an increase in the overall polymer hydrophilicity, strengthening polymer–water interactions and increasing the LCST [10], [12]–[14]. In addition the introduction of ionizable groups (–COO^−^) in the polymeric matrix results in pH-sensitive hydrogels that swell at pH values above the pK_a_ of the hydrophilic comonomers, and collapse below the pK_a_ [14], [15]. Additionally PNIPA is non-toxic [16] and its temperature-controlled on/off release mechanism has been has been widely investigated in biomedicine, and many other fields, for application in drug delivery systems [17]–[19], enzyme immobilization [20], [21] or gene carrier systems [22], [23].

On the other hand, taking hydrogels to the nanoscale may result in enhanced technological applications. Nanohydrogels combine the best properties of hydrogels (flexibility, hydrophilicity, great water absorption capacity) with the advantages of nanoparticles, allowing to obtain a better dispersion in the food packaging material and reduce the amount of bioactive compounds to be applied. In addition, the nanoscale dimensions and low hydrophobicity of PNIPA nanoparticles make them suitable for oral ingestion because they have a low immune response and can undergo glomerular filtration in the kidney [24].

Therefore, nanoencapsulation of bioactive compounds in PNIPA nanohydrogels can be performed for a number of purposes, such as protecting labile bioactive compounds from degradation during food processing, storage and usage, carrying molecules to their site of action, and controlling their release in active packaging systems. However, research on the potential application of PNIPA nanohydrogels in food active packaging is so far limited to our previous work [1], in which we reported the nanoencapsulation and controlled release of pimaricin in PNIPA nanohydrogels, and demonstrated its antimicrobial effectiveness employing a food model system.

To effectively incorporate nanoencapsulated pimaricin in antimicrobial active packaging systems for food applications, we must first gain a better understanding of the factors influencing the pimaricin release from PNIPA nanohydrogels. In this work we study the physicochemical properties of PNIPA nanohydrogels with different hydrophilicity grades, synthesized by adding different concentrations of acrylic acid (AA) as comonomer.

Previous studies were performed and analyzed using a one-factor-at-a-time approach. This methodology may be useful for comparative purposes since most of the reported works follow this experimental procedure [14], [25], [26]. However, one-factor-at-a-time approaches ignore interactions between factors and, since in real-food systems the pimaricin release from PNIPA/AA nanohydrogels is simultaneously influenced by both pH and temperature, these studies may lead to misleading conclusions. Therefore, in this work, we propose a simple experimental method to precisely determine the thermodynamic parameters associated with the collapse of PNIPA/AA nanohydrogels, induced by one (temperature) or two simultaneous factors (pH - temperature, methanol concentration - temperature). We also show how the environmental conditions and solvent in which the nanohydrogel is prepared determine its swelling/collapse behaviour, and therefore its capacity to encapsulate and release a bioactive compound to be applied in active packaging.

## Materials and Methods

### Materials

*N*-isopropylacrylamide (NIPA) (99%, stabilized) came from Acros Organics and was used as received. Acrylic acid (stabilized with hydroquinone monomethyl ether) for synthesis, *N*,*N*′-methylenebisacrylamide (NMBA) for synthesis and sodium bisulfite (NaHSO_3_) for analysis were from Merck. Isoparaffinic synthetic hydrocarbon (98%, Isopar™ M) was from Esso Chemie. Sorbitan sesquiolate (98%, Span™ 83) and PEG-40 sorbitol hexaoleate (98%, Atlas™ G-1086) were from Uniquema. Chloroform stabilized with ethanol, diethyl ether (stabilized with ∼6 ppm of BHT), methanol, sodium di-hydrogen phosphate anhydrous, tri-sodium citrate 2-hydrate and hydrochloric acid (37%) were from Panreac. TRIS buffer (tris-(hydroxymethyl)-aminomethane, extra pure) was from Scharlau.

### Synthesis of PNIPA/AA nanohydrogels

The microemulsion polymerization experiments were conducted in water-in-oil (W/O) systems. The W/O microemulsion composition was 58% aqueous phase (AP), 17% oil phase (OP) and 25% surfactant (ST). The AP consisted of 80% water and 20% monomers.

The mass ratios of NIPA to AA based on the monomers are shown in Table 1. To preserve the shape and size of the particles during handling we used NMBA as the cross-linker (CL) in the proportions indicated in Table 1. The OP consisted entirely of Isopar™ M, which was mixed directly with surfactants (ST) Atlas™ G-1086 (m_Atlas G-1086_/m_ST_ = 0.97) and Span™ 83 (m_Span 83_/m_ST_ = 0.03).

**Table 1:** Composition of the PNIPA/AA nanohydrogels.

Nomenclature	m_NIPA_/m_monomer_	m_AA_/m_monomer_	m_CL_/m_monomer_
PNIPA(3)	1.00	0.00	0.03
PNIPA(5)	1.00	0.00	0.05
PNIPA-10AA(3)	0.90	0.10	0.03
PNIPA-10AA(5)	0.90	0.10	0.05
PNIPA-20AA(3)	0.80	0.20	0.03
PNIPA-20AA(5)	0.80	0.20	0.05

NIPA: *N*-isopropylacrylamide.

AA: acrylic acid.

CL: cross-linker (*N*,*N′*-methylenbisacrylamide).

Both phases were solubilized then mixed in a 100 mL reactor thermostatized at 25°C, equipped with mechanical stirring and a thermal sensor. To eliminate oxygen, 10 min before the polymerization began (by adding the initiator at a ratio of m_NaHSO3_/m_monomer_ = 0.01) and during the entire reaction, the reaction medium was purged by bubbling nitrogen through it. The polymerization conversion was monitored via the temperature increase inside the glass reactor. The polymer was purified using selective precipitation with chloroform and diethyl ether. The pure polymer was dried overnight in an oven (50°C) and then ground in a colloid mill (IKA® -Werke GmbH & Co. KG).

### Measuring mean particle size and zeta potential

Zeta potential (Zp) and mean particle size (Z-average) were analyzed using a dynamic light scattering (DLS) measurement technique with Zetasizer Nano ZS (Malvern Instruments at 25°C. Nanohydrogel suspensions were prepared by dispersing PNIPA powder in distilled water (0.5 mg mL^−1^) with agitation for 3 hours at ambient temperature to allow proper swelling of the nanoparticles. Each analysis was performed in triplicate.

### Morphology observation by transmission electron microscopy (TEM)

For the examination of the morphology of the nanoparticles by TEM, 5 µl sample aliquots were absorbed to carbon-coated collodion film supported on 400-mesh copper grids, and negatively stained with 1% uranyl acetate. The grids were exhaustively visualized with a Jeol microscope (JEM-1400), operated at 80 kV.

### Study of the gel phase transition

The phase transition phenomenon of the polymer suspensions (12.5 mg mL^−1^) was examined using two methods:

Optical density (OD). The phase transition was examined by measuring the OD of polymer suspensions at 500 nm using a spectrophotometer with a Peltier temperature control module (Beckman Coulter Inc.) at temperature intervals from 15 to 50°C with increments of 2°C between each measurement.

Differential scanning calorimetry (DSC). DSC scans (Micro DCS III, Seratam) of nanohydrogel suspensions were performed from 10 to 50°C with the reference ampule containing distilled water. Scans were run at 0.25°C min^−1^.

For the study of the temperature effect nanohydrogel suspensions were prepared dispersing PNIPA/AA powder in distilled water (pH 5.5) at a concentration of 12.5 mg mL^−1^, by agitation during 3 hours at ambient temperature to allow a proper swelling of the nanoparticles.

On the other hand, for the study of the combined effect of pH and temperature the range of pH assayed was from 2 to 8. The following buffers were used to disperse PNIPA/AA powder in the same concentration aforementioned: citrate-HCl buffer (pH 3 to 6), Tris–HCl (pH 8) and phosphate-HCl (pH 2 and 7), by agitation during 3 hours at ambient temperature to allow a proper swelling of the nanoparticles.

For the study of the combined effect of methanol and temperature the volume fraction of methanol in distilled water (v/v) assayed was from 0 to 1. PNIPA/AA powder was dispersed, in the same concentration aforementioned, in the different methanol/distilled water mixed solutions during 3 hours at ambient temperature to allow a proper swelling of the nanoparticles.

Each analysis was performed in duplicate.

### Data fitting and statistical analysis

The differences between the results were evaluated using analysis of variance (ANOVA) followed by Bonferroni post-tests for multiple comparisons using GraphPad Prism™ 5 (GraphPad Software Inc.).

In systems involving two variables, plotting, data fitting, parametric estimation and significance tests, both for parameters and models, were performed with SigmaPlot 11.0 (Systat Software, Inc.). Mathematica 7 (Wolfram Research, Inc.) was used for models describing the relationship between more than two variables.

## Results and Discussion

### Mean particle size and zeta potential

**Figure 1:**
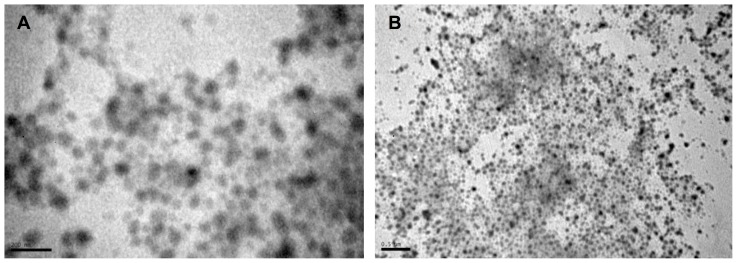
Representative TEM micrographs of PNIPA nanohydrogel. Scale bar of the image A is 200 µm.

Figure 1 shows the TEM micrographs of PNIPA nanoparticles. It has been clearly observed that nanohydrogels are well dispersed and spherical in shape. Nanoparticle sizes which can be observed in TEM micrographs are lower than those obtained by DLS (Figure 1). The samples was dried to be analysed by TEM while the nanoparticles was swollen in water to be measured by DLS. Values obtained by this last technique are referred to the hydrodynamic radius of a solvated molecule.

**Figure 2:**
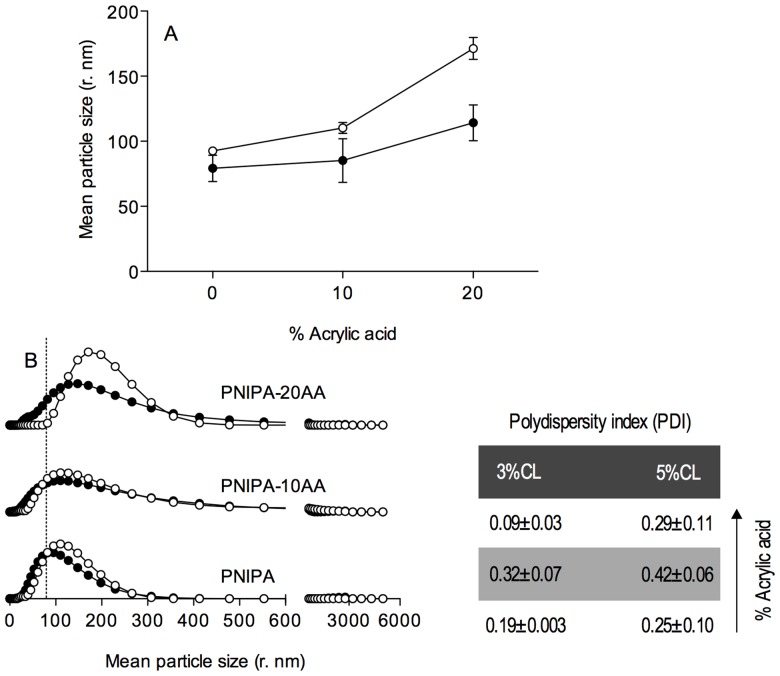
Mean particle size (A) and size distribution (B) of PNIPA/AA nanohydrogels. Nanohydrogels with 3% (○) and 5% (•) of the cross-linking (CL) degree were previously swelled and measured in distilled water (pH 5.5) at 25°C. The table with the polydispersity index (PDI) values indicates the nanoparticles size dispersion.

The Z-average (r, nm) obtained by DLS ranged from 79 to 171, with a polydispersity index (PDI) always below 0.5 (Table inset Figure 2). The PDI is an indication of variance in the sample; a low PDI (usually less than 0.2) indicates that the sample is monodispersed. All samples had a single population, and the PDI values showed a limited variation in particle size (a narrow distribution). The latter was particularly noticeable for the nanohydrogels without acrylic acid (PNIPA) independently of the CL-content. Low PDI indexes were also observed for the PNIPA-20AA nanohydrogels and slightly higher indexes, but below 0.5, were found for PNIPA-10AA.

Figure 2A shows that nanogels with 3% CL had larger sized particles than those with 5% CL for all conditions tested. In addition, regardless of the CL percentage, increasing the AA-content increases the Z-average.

A two-way ANOVA (at a 95% confidence level) of the Z-average variation shows that the effects of both the CL-degree and AA-content were statistically significant (*p*<0.0001).

Moreover, the interaction between these two factors was also significant (*p* = 0.0093), i.e., the effect of CL-content on the Z-average changed as a function of the AA-content.

Therefore, the differences in swelling degree between 3% and 5% CL nanoparticles became higher as the AA-content increased.

Since the nanoparticles were allowed to swell for 3 h in the solvent (water) at 25°C, these effects on the Z-average values are an indication of the swelling properties of the nanohydrogels.

The swelling degree is considered to be the result of the balance between two opposite forces, the osmotic pressure, which tends to expand the gel, and the elasticity of the polymer, which shrinks the gel network. Elasticity is, in turn, strongly influenced by the cross-linking degree of the gel matrix.

In our experimental conditions, increasing the CL concentration from 3% to 5% led to a decrease in the particle size. In agreement with Çaykara et al. [17], and Otake et al. [27], increasing the cross-linking points within a gel structure hinders the formation of hydration structures around these positions, which decreases the overall water absorption. That is, decreasing the CL concentration promotes swelling and thus the gel elasticity.

Figure 2B also indicates that copolymerization of PNIPA with AA increases the particle size. As explained by Nayak et al. [26], this behaviour may be partially caused by the coulombic repulsion, that arises as a result of the pH-dependent ionization of acrylic acid. The pK_a_ of AA is approximately 4.35 [28], however the calculation of their pK_a_ after polymerization with NIPA polymer is a difficult task as we could not determine the structural repeating unit in cross-linked NIPA polymers and therefore we were unable to obtain reliable data regarding the groups around -COOH moieties that might influence its pK_a_. Nonetheless, since after polymerization AA looses the double bond and turns into isobutyric acid, a better approach may be to consider the pK_a_ of isobutyric acid (4.86). Taking into account this value, at the pH of the solution (5.5), around 81.4% of the carboxylic groups would be ionized (R–COO^−^), increasing the particle swelling even more (the greater the number of charged groups, the greater the hydration capacity [29]). In addition, the higher the ionization, the higher the osmotic pressure by counter ions within the nanohydrogel, which contributes to the expanding force giving a more swelled nanoparticle [30].

The described effects appear to be more intense in 3% CL gels, which at 20% AA showed a size increment (relative to the gel size at 0% AA) equal to 1.3-fold the size increment for 5% CL gels. The simultaneous increase in AA-content and CL-degree may facilitate the positioning of the hydrogen-bond forming groups (AA and NIPA chains) at the right distances to permit the formation of additional interaction points resulting in more compressed gel structures.

**Figure 3:**
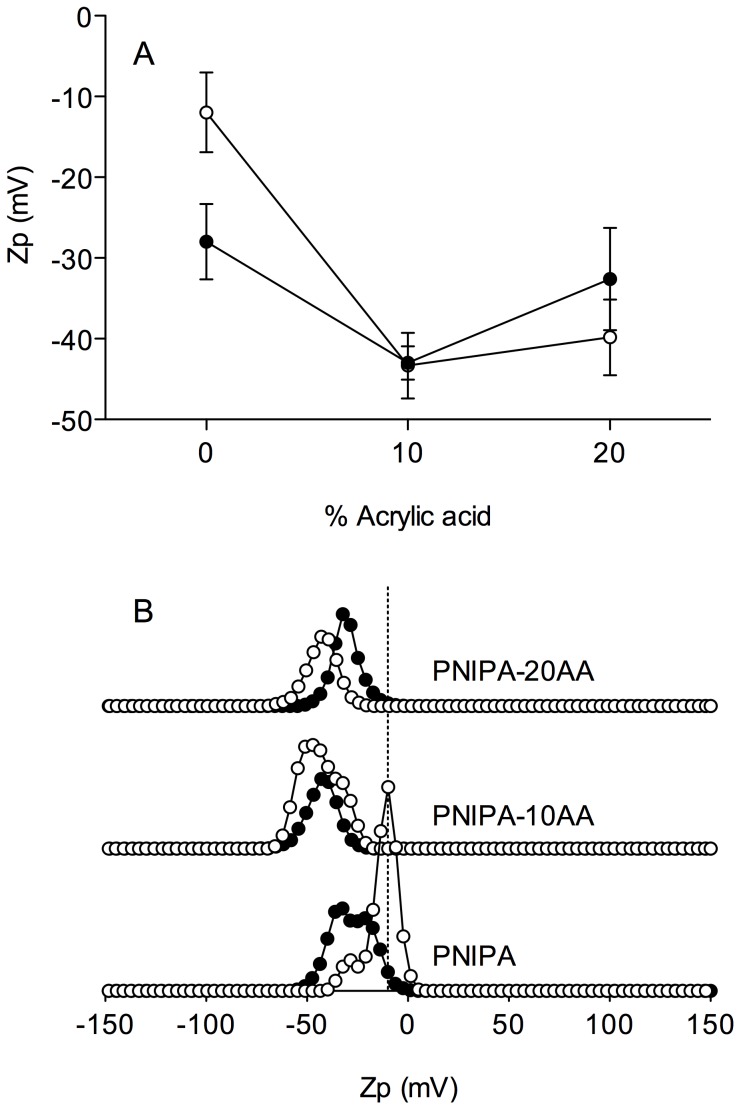
Mean zeta potential (Zp) (A) and zeta potential distribution (B) of PNIPA/AA nanohydrogels. Nanohydrogels with 3% (○) and 5% (•) of the cross-linking (CL) degree were previously swelled and measured in distilled water (pH 5.5) at 25°C.

The zeta potential of a nanoparticle is commonly used to characterize the surface charge property of nanoparticles. It reflects the electrical potential of particles and is influenced by the composition of the particle and the medium in which it is dispersed [31]. Figure 3B shows the zeta potential distribution for the synthesized PNIPA nanohydrogels. The effect of the CL-degree was only significant (*p*<0.01) for the PNIPA homopolymers (Figure 3A).

In addition, all samples, including the nominally electrically neutral PNIPA(3) and PNIPA(5) gels, have a negative zeta potential at pH 5.5.

PNIPA(3) shows a narrow distribution with a modal peak at a value of −9.76 mV but also a second small peak in the distribution at a lower zeta potential (−28.47 mV). PNIPA(5) shows at least two poorly-separated populations with a more negatively charged zeta potential than PNIPA(3) gels (main peak), despite neither of them having ionized groups. In contrast, mono-modal particle size distributions were obtained with similar particle sizes for PNIPA(3) and PNIPA(5) gels, 92.65±0.11 nm and 79.19±10.06 nm respectively.

A similar finding was reported for neutral nanoparticles of PNIPA copolymerized with poly (ethylene glycol). According to Tauer et al. [32], the slightly negative Zp of PNIPA gels might be explained by the assumption of a contact potential as described by Coehn's rule [33]. Accordingly, there is an electric potential between two materials with different permittivity so that the material with the lower dielectric constant carries the negative charge [32]. It is currently unclear why the Zp profile changes so dramatically when the CL concentration of the PNIPA homopolymers (Figure 3) is modified but it might be related to the influence of the elasticity of soft particles on permittivity.

Additionally it is reported, that even in the absence of ionizable functional groups, neutral surfaces might acquire a surface charge through the adsorption of anions from the solution [34]. If so, anions would have less tendency to approach the surface of PNIPA-co-AA nanohydrogels due to probable electrostatic repulsion. Although none of the reactants used to synthesize the PNIPA nanohydrogels is anionic in nature, the presence of impurities that contain acidic functional groups in the commercial chemicals employed cannot be ruled out.

PNIPA copolymerized with AA (PNIPA-co-AA) nanohydrogels always had a lower Zp value than PNIPA nanohydrogels without AA. The pH and ionic strength in solution are two main factors that affect the zeta potential of charged particles. The pH determines the extent of protonation of functional groups on the particle surface, while elevated ionic strength compresses the charged layer surrounding the particles [35]. The zeta potentials measured in distilled water are a good approximation of the surface potential of the investigated materials.

As already mentioned at the solution pH (5.5), around 93% of the AA would be ionized thus promoting a great increase in the surface negative charge of the PNIPA-co-AA nanohydrogels. In these cases, the contribution of the CL to the surface charge is comparatively low and hence can be neglected.

Although Figure 3A indicates a sharp fall on Zp values (become more electronegative) when PNIPA nanogels were cross linked with AA, no great differences on Zp were observed when the AA content increased from 10 to 20%. This may be explained assuming that the charge density (or charge concentration) on the surface of the nanoparticles copolymerised with 10 and 20% of AA were very similar. The charge density might be roughly estimated using the equation proposed by Serrano-Medina et al. [36]: 
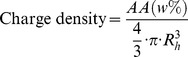
(1)where *R_h_* is the hydrodynamic radius.

Results obtained using Equation 1 are shown in Table 2. Although the charge density values estimated for nanohydrogels with different %CL were fairly different, when the %CL of the PNIPA-co-AA nanogels were kept constant an increase on the AA concentration from 10 to 20% resulted in a far lower variation in charge density, which agrees with the zeta potentials measurements”.

**Table 2:** Charge density (w% µm^−3^×10^3^).

AA-content	3% CL	5% CL
10	1.78	3.86
20	0.95	3.21

AA: acrylic acid.

CL: cross-linker (*N*,*N′*-methylenbisacrylamide).

### Thermodynamic properties associated with the nanohydrogel collapse

At the beginning of paper we explained how PNIPA/AA hydrogels are able to undergo reversible volume phase transitions in response to environmental factors. In the following sections we are going to study the influence of these factors on the nanohydrogel collapse and how the composition determines the thermodynamic properties associated with the nanohydrogel collapse.

#### Temperature-induced collapse

**Figure 4:**
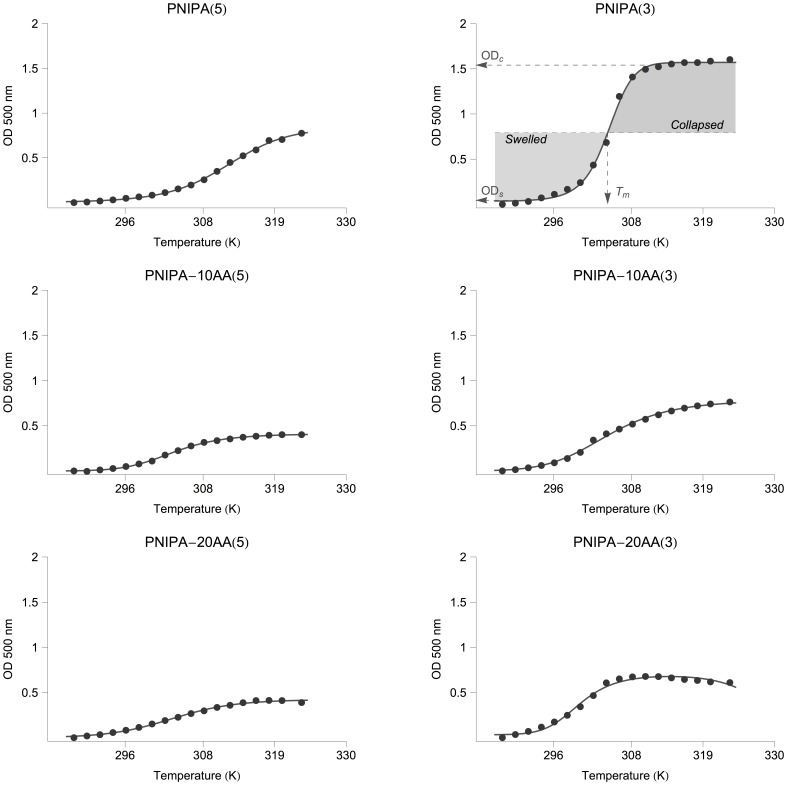
Optical density (OD) variation of PNIPA/AA nanohydrogel as a function of temperature (K). Nanohydrogels synthesized with 3% and 5% of cross-linking degree (CL) were previously swelled in distilled water (12.5 mg mL^−1^) at 25°C. The lines are the fitting curves generated from Equation 13. The meaning of the most relevant parameters for defining a collapse curve of a PNIPA nanohydrogel exposed to increasing temperatures is graphically illustrated in the subfigure PNIPA(3): *T_m_* represents the lower critical solution temperature (LCST), *OD_s_* and *OD_c_* are the characteristic optical density values in the swelled state and the collapsed state, respectively.”

Figure 4 shows the OD variation of PNIPA/AA nanohydrogels previously swollen in distilled water (pH 5.5), as a function of the temperature. Nanohydrogels of PNIPA homopolymer showed a sharp volume phase transition, which is usually related to the hydrophilic/hydrophobic balance of the apolar side groups shifting to the hydrophobic side, which leads to a rapid dehydration of the polymer as the temperature increases above the LCST [37]. However, PNIPA-co-AA nanohydrogels displayed a more subtle transition with a diffuse LCST. It has been demonstrated that the physical properties of PNIPA nanohydrogels, such as the LCST, can be modulated by copolymerization with hydrophilic monomers [12], [38].

Thus, the observed differences should be related to the characteristics that acrylic acid confers to the hydrogel. For instance, in PNIPA-co-AA nanohydrogels, new hydrogen bonds between water and acrylic acid are added to those already established within the PNIPA structure, which hinders the dehydration of the polymer chains and acts to expand the collapsed structure [37].

In addition, the ionized carboxyl groups of AA (existing at pH 5.5) are soluble enough to counteract the aggregation of the hydrophobic temperature-sensitive elements [37], [39]. Furthermore, electrostatic repulsion between –COO^−^ groups in AA or the formation of hydrogen bonds with other PNIPA components might lead to a more hydrated collapsed state, or even prevent the collapse of the structure.

Other authors have also suggested that a stronger hydrogen bond interaction could explain the change in LCST behaviour observed with PNIPA-co-AA nanohydrogels. Chen et al. [40] proposed that polymer nanoparticles collapse at higher temperatures due to the higher energy required to destroy the newly-formed hydrogen bonds. The same behaviour was also observed with other hydrophilic copolymers. Milašinović et al. [14] observed a similar trend when the hydrophilicity degree was increased by augmenting the percentage of itaconic acid. In this case, the PNIPA homopolymer showed a clear LCST at 34.2°C, with a sharp phase transition that became less pronounced for hydrogels with 5 and 10% itaconic acid, and which practically disappeared when the itaconic acid concentration was increased up to 15%.

Should be noted that negative surface charge of the PNIPA(5) homopolymer observed in the previous section lead to a similar behaviour to that produced by the presence of AA resulting in a more subtle transition than the PNIPA(3) homopolymer, which had a surface charge close to neutrality.

With respect to the influence of the CL-degree, the profiles of OD variation with temperature (Figure 4) agree with the explanation offered in the previous section. The increase in the rigidity of the nanoparticles due to the increment in the CL-degree leads to smoother volume changes associated with thermal transitions. Coughlan and Corrigan [41] suggested that the increase in the CL-content could also lead to an increase in the overall hydrophilicity of the PNIPA hydrogels.

Since the swelling/collapse behaviour of nanohydrogels determine their efficiency as carriers in smart delivery systems, comparing between different nanohydrogels means that the thermodynamic changes associated with the phase transition need to be determined precisely. Despite this critical parameters such as the LCST are often estimated from thermal transition curves using graphical methods, which do not provide confidence intervals and give little information on the physical changes accompanying the collapse [40], [42], [43].

Nonetheless, the simplicity of the collapse transition and its reversibility make it possible to make a detailed thermodynamic description of the collapse process by analogy to the van't Hoff analysis of the unfolding of globular proteins [44]–[46]. A similar parallelism with the protein unfolding was also proposed by Alf et al. [47] and Tiktopulo et al. [48] for calculating the enthalpy changes during the thermally-induced coil to globule transition of PNIPA chains and cross-linked gels, and by Kato [49] for analyzing the effect of pressure on PNIPA gels using the Clausius-Clapeyron relation, which they discuss in terms of the thermodynamic theory of the reversible pressure-temperature denaturation of proteins. In particular, Tiktopulo et al. [48] pointed out the fact that PNIPA is a polyvinyl polymer that is a chemical isomer of poly-leucine and therefore PNIPA gels could be treated as synthetic models for the studying of protein stability.

Basically, the proposed approach consist on monitoring the hydrogel collapse by measuring any spectroscopic parameter (in our case, the OD) and then plotting the experimental data (Figure 4 shows a typical collapse transition) OD values characteristic of the swelled state (*OD_s_*) and of the collapsed state (*OD_c_*) can be obtained from the bottom and top plateau of the curve. At any temperature (*T*) in the transition, the hydrogel would be collapsed at a certain level. Assuming a simple two-state equilibrium [46] (Swelled 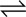 Collapsed), a partially collapsed hydrogel is mathematically equivalent to considering the coexistence of a fraction of swelled hydrogel (*f_s_*) in equilibrium with a fraction of collapsed hydrogel (*f_c_*). Therefore, at any point in the collapse curve: 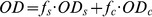
(2)since, 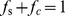, 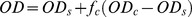
(3)and the fraction of nanohydrogel collapsed at any point: 
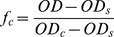
(4)


Thus, the apparent equilibrium constant of collapse (*K_c_*) is: 
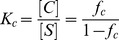
(5)and from this equation, 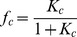
(6)


On the other hand, at equilibrium (when 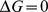), the standard free energy change (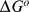) accompanying the collapse at a temperature *T* is related to the equilibrium constant (*K_c_*) between the swelled and the collapsed states by: 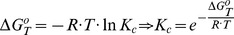
(7)where *R* is the gas constant (8.314 J mol^−1^ K^−1^) and *T* is the absolute temperature (K). In addition, from the Gibbs free energy definition: 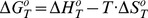
(8)where 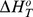 and 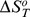 are the enthalpy and entropy changes accompanying the collapse at temperature *T*.

When the hydrogel collapses (i.e., changes its thermodynamic state), a molar heat capacity change (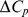) is observed due to the solvent molecules being reconstructed around exposed moieties of the hydrogel [10], [27], [50]. Assuming that 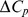 between the swelled and collapsed states of the gel is independent of the temperature in the experimental domain [51], the temperature dependences of 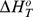 and 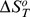 can be calculated according to the Kirchhoff's law: 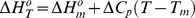
(9)

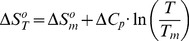
(10)where 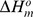 and 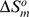 are the enthalpy and entropy changes at the midpoint of the collapse transition, i.e., when 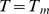 (being Tm the LCST for the nanohydrogel).

Substituting values of Equations 9 and 10 into Equation 8 we get: 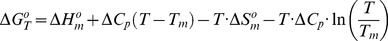
(11)


At 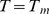 we have the same concentration of swelled and collapsed gel, so 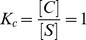 (from Equation 5), 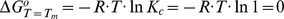 (from Equation 7), and 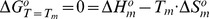 (from Equation 8).

Therefore 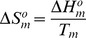. Substituting 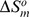 into Equation 11 we get: 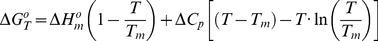
(12)


Finally, rearranging Equations 3, 6, 7 and 12 one obtains: 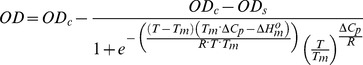
(13)


Figure 4 and Table 3, show the fittings of Equation 13 to the experimental data. The obtained best-fit models provided significant values for all the parameters except 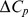.

**Table 3:** Parameters generated from fitting the Equation 13 to experimental data of optical density variation in PNIPA/AA nanohydrogel suspensions as a function of temperature.

Nanohydrogel	*T_m_* (K)	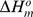 (KJ mol^−1^)	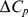 (KJ mol^−1^ K^−1^)	r^2^
PNIPA(3)	304.34±0.27^a^^,^ α	382.16±44.95^a^^,^ α	23.28±24.89^a^^,^ α ^,^ n.s.	0.9990
PNIPA(5)	311.67±0.62^a^^,^ β	172.34±9.79^a^^,^ β	3.19±4.06^a^^,^ α ^,^ n.s.	0.9990
PNIPA-10AA(3)	305.08±4.85^a^^,^ α	143.29±93.44^b^^,^ α ^,^ n.s.	−5.59±8.56^ab^^,^ α ^,^ n.s.	0.9987
PNIPA-10AA(5)	303.59±0.92^b^^,^ α	190.33±28.89^a^^,^ α	−6.66±7.15^a^^,^ α ^,^ n.s.	0.9996
PNIPA-20AA(3)	300.41±0.58^a^^,^ α	239.07±47.01^ab^^,^ α	−17.89±3.23^b^^,^ α	0.9966
PNIPA-20AA(5)	303.27±0.89^b^^,^ α	157.51±15.38^a^^,^ α	0.23±6.04^a^^,^ α ^,^ n.s.	0.9982

Values reported are the means±standard deviation (*n* = 2).

a,b,c Statistically significant differences (*p*<0.05) between gels with different acrylic acid content for the same cross-linking degree.

α,β Statistically significant differences (*p*<0.05) between gels with different cross-linking degree for the same acrylic acid content.

n.s. Parameter not significant (*p*>0.05).

In addition, high-adjusted r^2^ values were obtained, which indicate that Equation 13 is a good predictor of the swelling-collapse transition. However, despite the obvious differences between the swelling-collapse kinetics, we found almost any significant difference in the *T_m_* between nanohydrogels with different composition. In fact, PNIPA(5) was the sole nanohydrogel with a significantly different (*p*<0.05) *T_m_* value. At this respect, Lin et al. [13] pointed out that different PNIPA-co-AA hydrogels show a broad range of volume phase transition temperatures due to the presence of macroscopic and microscopic volume phase transitions [52], [53]. Although both transitions can occur, only the first one (major particle shrinking) being clearly observed with the measurements of UV–Vis transmittance [13]. Thus, the observed transition-temperature differences might be lower than expected.

**Figure 5:**
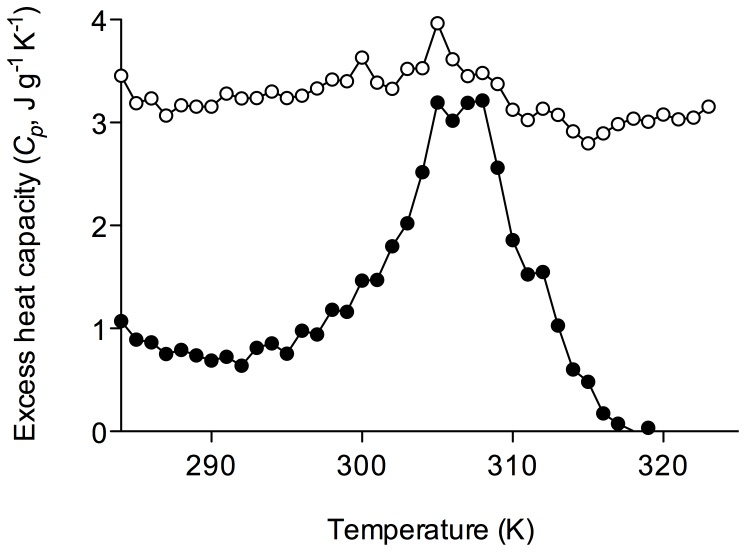
Differential scanning calorimetry (DSC) analysis of PNIPA/AA nanohydrogels. PNIPA(5) (•) and PNIPA-20AA(5) (○) nanohydrogels were previously swelled in distilled water (12.5 mg mL^−1^) at 25°C.

On the other hand, the volume phase transition from the swelled to the collapsed state should be an endothermic process involving a great heat transition due to the formation of hydrophobic interactions [27] and the disappearance of the polymer–water interactions [27], [54]. Therefore, the effect of incorporating AA as comonomer in the NIPA polymer could also be studied by DSC [10], [54], which also allows the specific heat capacity (*C_p_*) of even small quantities of nanohydrogel to be measured directly. For the sake of simplicity, these studies were limited to PNIPA(5) and PNIPA-20AA(5), two nanohydrogels that clearly illustrate the effect of introducing a hydrophilic comonomer. The obtained heat flow curves are shown in Figure 5. The PNIPA homopolymer displays an endothermic peak, which could be attributed to its LCST (around 33°C). However, the DSC profile of the PNIPA-20AA(5) polymer reveals that either there were no peaks or their intensity was very low, which agrees with the results obtained with optical density measurements (Figure 4). Eeckman et al. [12] and Feil et al. [55] observed similar behaviour, that is, that the width of the transition endothermic peak increased when an AA comonomer was introduced into a PNIPA polymer.

**Figure 6:**
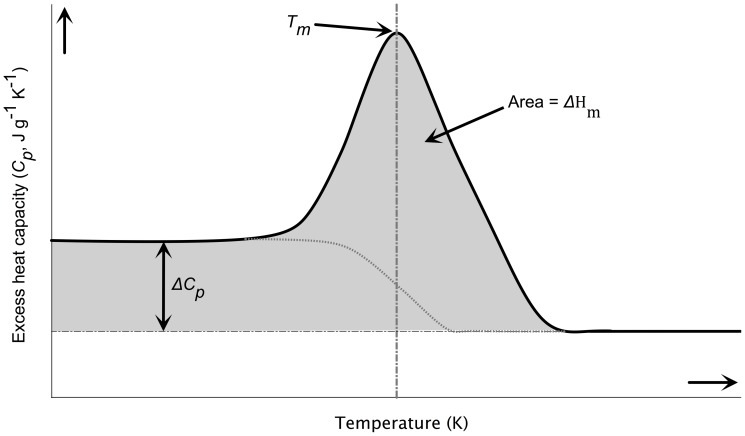
Example of differential scanning calorimetry (DSC) analysis of a PNIPA/AA nanohydrogel aqueous solution. This figure was adapted from Bruylants et al. [43]. *T_m_* corresponds to the lower critical solution temperature (LCST), 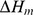 is the enthalpy changes at the midpoint of the collapse transition, i.e., when 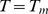 and 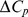 is the heat capacity change.

As mentioned above, the DSC curves can be used to calculate the heat capacity change (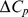) when the hydrogel collapses as the baseline shifts. In addition, the amount of energy needed for the transition, i.e., the enthalpy (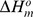) can be obtained by integrating of the *C_p_* vs. *T* curve (Figure 6). The 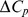 values calculated from DSC curves (Figure 5) were −0.85 J g^−1^ K^−1^ for PNIPA(5) and −0.27 J g^−1^ K^−1^ for PNIPA-20AA(5). However, we could not compare these values with those obtained with Equation 13, since the 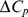 values shown in Table 3 are not significant (*p*>0.05) for PNIPA(5) or PNIPA-20AA(5). The 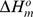 values obtained for the PNIPA(5) and PNIPA-20AA(5) nanohydrogels were 45.06 J g^−1^ and 15.66 J g^−1^ respectively.

Finally, although the van't Hoff enthalpy (Table 3) may be different from the calorimetric one, because the calorimetric enthalpy measures the total enthalpy change including the contribution from all processes, and is determined independently of any model while the corresponding van't Hoff enthalpy assumes a simple two-state transition [44], the ratio between these two enthalpies can provide an estimation of the molecular weight of our polymers. The calculated molecular weights were 3824.67 g mol^−1^ for PNIPA(5) and 10058.11 g mol^−1^ for PNIPA-20AA(5).

#### Simultaneous temperature and pH-induced collapse

In the previous sections we saw how introducing hydrophilic comonomers into PNIPA nanohydrogels varies their response to increasing temperatures due to the presence of ionized –COO^−^ groups, which prevents the nanohydrogels from collapsing. However the ionization of these groups is determined by the pH value of the medium in which the nanohydrogels are intended to be applied. Thus, a new set of experiments was performed to evaluate the combined effect of pH and temperature on the collapse of the PNIPA/AA nanohydrogels.

**Figure 7:**
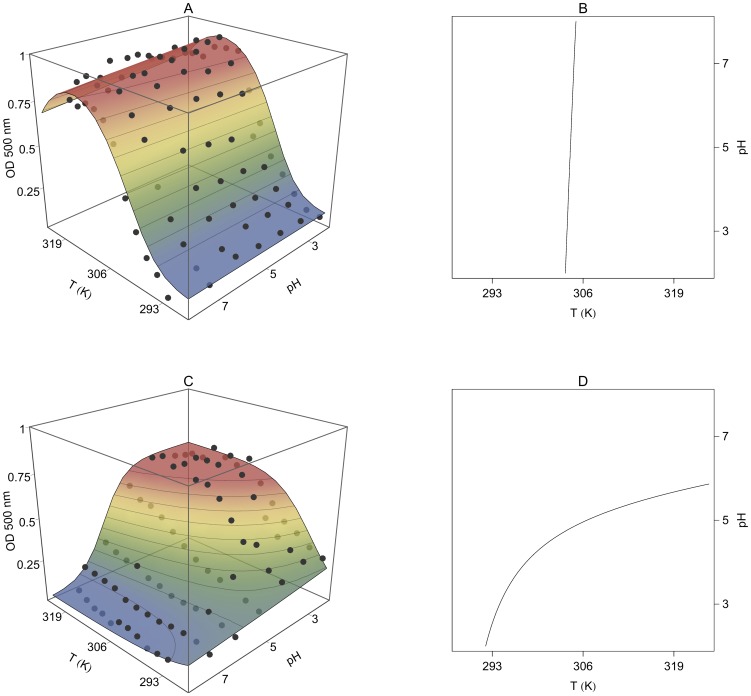
Optical density (OD) variation of PNIPA/AA nanohydrogels as a function of pH and temperature. PNIPA(5) (A) and PNIPA-20AA(5) (C) nanohydrogels were previously swelled in distilled water (12.5 mg mL^−1^) at 25°C. The surface represents the fitting of the experimental data with the Equation 19. Figures B (PNIPA(5)) and D (PNIPA-20AA(5)) show pH-T phase diagrams, in which the critical line marks the temperature and pH conditions at which the nanohydrogel begins to undergo the volume phase transition.

Figure 7 shows the observed OD variation as a function of temperature and pH. The responses of the gels with or without AA were very different. PNIPA-20AA(5) nanohydrogel (Figure 7C) was more swollen at high pH values, and the temperature had practically no effect. At pH values above the estimated pK_a_ of copolymerized AA (4.86), their carboxylic groups are ionized, resulting in an intense electrostatic repulsion, which prevents the hydrophobic interactions responsible for the gel collapsing with increasing temperatures. However, in acidic media, below the pK_a_, the carboxylic groups are protonated, and hydrogen bonds between the –COOH groups of AA and –CONH groups of the NIPA can be formed promoting the collapse of the gel [15]. Therefore, under the pK_a_, the lower the pH, the greater the nanohydrogel collapse, i.e. the OD increases, as well as the influence of the temperature. However, the pH had no influence on the PNIPA(5) nanohydrogel (Figure 7A) due to the absence of ionizable groups in the homopolymer. Díez-Peña et al. [56], also found these different behaviours in PNIPA nanohydrogels copolymerized with methacrylic acid, and Yoo et al. [57] in hydrogels of PNIPA copolymerized with AA.

In this case, to model the hydrogel transition we must consider that the observed 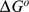 accompanying the collapse would be the sum of two contributions: pH change and temperature. The followed approach was based on the linear extrapolation method proposed by Pace and Shaw^56^ to evaluate the effect of a chemical denaturant on 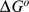 during the protein unfolding: 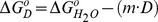
(14)where 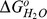 is the 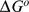 in the absence of denaturant and m is a measure of the dependence of 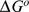 on the denaturant concentration (*D*).

Despite being an empirical relationship that does not have a theoretical basis, the model is widely used as it provides good fits for a large number of chemical denaturants. In this work we adapted the extrapolation method to reflect the effect of pH. In this case, we cannot obtain a 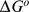 value in the absence of denaturant as every single media would have a pH value. However, we can make an analogy using a reference pH (*pH_r_*) as the “absence of denaturant”, which for us is the pH of the nanohydrogel suspension in distilled water (5.5). Similarly, the “denaturant concentration” would be the difference between the actual pH and the reference pH: 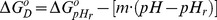
(15)


An additional term (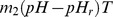) was included to reflect the possible interaction with temperature, whereby Equation 15 would become: 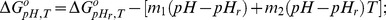
(16)


As, at any temperature, the 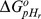 should coincide with the 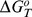 described in Equation 12, substituting its value in Equation 15, the observed 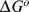 as a function of pH and temperature would be: 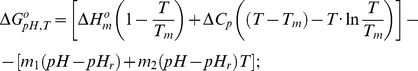
(17)and Equation 7 would be: 
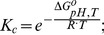
(18)


Finally, rearranging Equations 3, 6, 17 and 18 we obtain: 
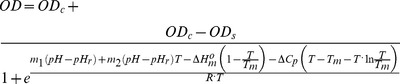
(19)


Equation 19 provided a good fit of the experimental data and the parameters generated (Table 4) were consistent with the theory, i.e., the parameters related to the pH influence (*m_1_* and *m_2_*) are not significant (*p*>0.05) for the nanohydrogel without AA (PNIPA(5)) and became significant (*p*<0.05) once AA was introduced into the nanohydrogel (PNIPA-20AA(5)). The drop observed in the response surface for the PNIPA(5) nanohydrogel at the higher temperatures (Figure 7A) is probably due to the fact that once the gel is fully collapsed at high temperatures, it begins to aggregate and precipitate, resulting in an increase in transmittance, i.e., a decrease in the OD value.

**Table 4:** Parameters generated from fitting the Equation 19 to experimental data of optical density variation in PNIPA/AA nanohydrogel suspensions as a function of temperature (T, K) and pH.

	PNIPA(5)	PNIPA-20AA(5)
*T_m_* (K)	304.31±2.28^a^	315.05±1.99^b^
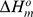 (KJ mol^−1^)	135.92±58.12^a^	64.76±16.10^a^
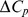 (KJ mol^−1^ K^−1^)	−12.97±3.02^a^	1.85±1.24^b^^,^ n.s.
*m_1_*	0.53×10^−3^±2.40×10^−3^ a ^,^ n.s.	42.96×10^−3^±6.62×10^−3^ b
*m_2_*	−2.11±7.77^a^^,^ n.s.	−150.15±22.73^b^
r^2^	0.9842	0.9818

Values reported are the means±standard deviation (*n* = 2).

a,b Statistically significant differences (*p*<0.05) between nanohydrogels.

n.s. Parameter not significant (*p*>0.05).

In addition, the *T_m_* values calculated with Equation 19 reflect the effect of AA on the collapse of the PNIPA/AA nanohydrogel better than those calculated with Equation 13, perhaps because we used a larger number data here that include measurements in a basic and an acidic medium which allowed us to calculate the parameters more accurately. Díez-Peña et al. [58] appointed that PNIPA copolymerized with methacrylic acid hydrogels with significant NIPA content lose the LCST transition in a basic medium but regain it in lower pHs. This behaviour can be attributed to the presence of ionized –COO^−^ groups at basic pHs, which prevents the nanohydrogels from collapsing as explained above. Thus, we found significant differences (*p*<0.05) between PNIPA(5) and PNIPA-20AA(5) (Table 4), increasing its *T_m_* from 31.31 to 42.05°C when we introduced 20% of AA into the nanohydrogel. These values are in accordance with those observed by other authors who studied PNIPA/AA hydrogels [15], [16], [40].

Figures 7B and 7D show pH-T phase diagrams in which the critical line marks the temperature and pH conditions at which the nanohydrogel begins to undergo the volume phase transition.

#### Simultaneous temperature and methanol-induced collapse

**Figure 8:**
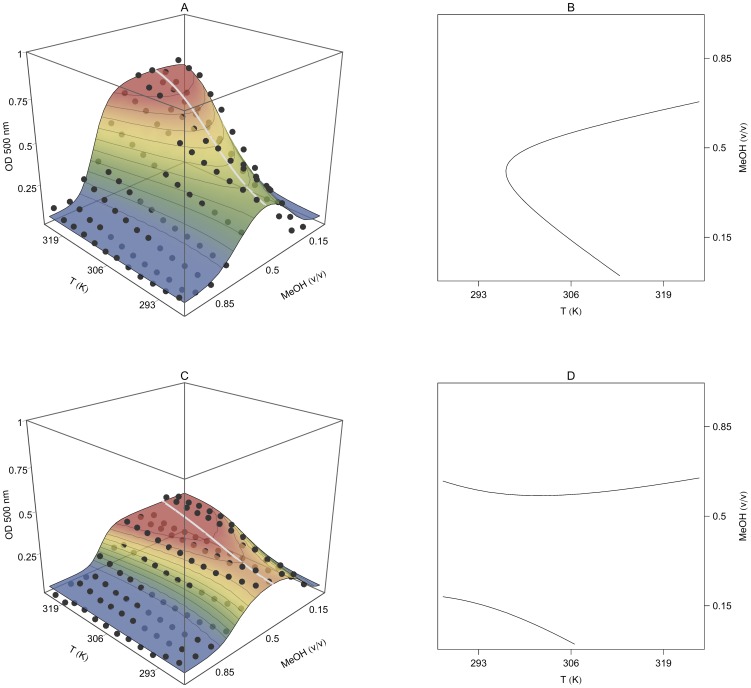
Optical density (OD) variation of PNIPA/AA nanohydrogels as a function of the methanol volume fraction and temperature. PNIPA(5) (A) and PNIPA-20AA(5) (C) nanohydrogels were previously swelled in distilled water (12.5 mg mL^−1^) at 25°C. The surface represents the fitting of the experimental data with the Equation 23. Figures B (PNIPA(5)) and D (PNIPA-20AA(5)) show MeOH-T phase diagrams in which the critical line marks the temperature and MeOH conditions at which the nanohydrogel begins to undergo the volume phase transition. The grey line in Figures A and C, calculated with Equation 27, represents the critical methanol concentration at which the tendency in the nanohydrogel collapse changes.

Previous studies by other authors have evaluated the effect of organic solvents on the collapse of PNIPA hydrogels. These studies revealed a phenomenon known as cononsolvency, i.e., pure water and pure alcohol are both good solvents for PNIPA, but mixtures of the two, over a certain concentration range, form a poor solvent [59]. Cononsolvency is produced by the formation of clathrate structures consisting of water molecules that encapsulate alcohol molecules due to local ordering of the water structure [60], [61]. These clathrate structures are stable up to a critical alcohol to water ratio (always in the water-rich region) at which there is no longer enough water to provide clathrate structures, and the alcohol molecules can come into contact with each other [61], allowing the PNIPA nanohydrogel to swell again. Our results (Figure 8) clearly illustrate this behaviour.

Based on these considerations, and using the same assumptions as for the temperature and pH-induced collapse, the model proposed by Pace and Shaw [62] (Equation 14) was modified to describe the effect of methanol on the Δ*G^o^* during the nanohydrogel collapse: 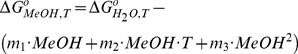
(20)where 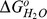 is the 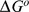 in the absence of methanol, and *m_i_* parameters give a measure of the dependence of 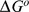 on the methanol concentration (MeOH, expressed in terms of volume fractions, v/v). As in the pH-T model, *m_2_* reflects the possible interaction with temperature. In addition an extra term (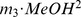) was included to reflect the effect of the clathrate formation.

Again, since at any *T* value Δ*G^o^* in the absence of methanol (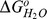) coincides with the 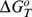 described in Equation 12, substituting its value in Equation 20, the observed 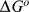 as a function of the MeOH concentration and temperature would be: 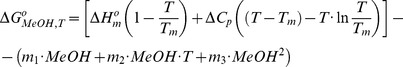
(21)and Equation 7 should be modified as follows: 
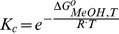
(22)


Finally, rearranging Equations 3, 6, 21 and 22 we obtain an equation that relates the simultaneous effect of the temperature and MeOH concentration on the collapse of PNIPA/AA nanohydrogels: 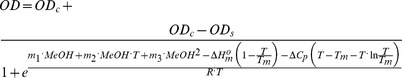
(23)


In this case, we can also calculate, for each temperature, the critical methanol to water ratio at which clathrates are no longer stable and the nanohydrogel begins to swell again. The contribution of the methanol to the collapse process is given by the relationship: 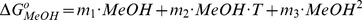
(24)


At any temperature the critical methanol/water ratio is determined by the optimum of this curve (Equation 24). Since the partial derivative with respect to the methanol volume fraction at the optimum would be 0: 
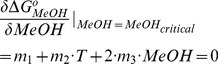
(25)we can calculate the critical methanol/water ratio as a function of temperature: 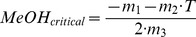
(26)


This two-variable relationship can be represented in 3D-space (*T*, *MeOH*, *OD*) as a parametric curve (grey line in Figures 8A and 8C): 
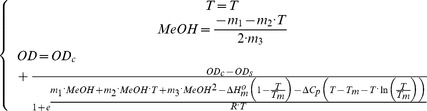
(27)


Figure 8 shows the fitting of Equation 24 to the experimental OD variation as a function of the volume fraction of methanol (v/v) and temperature. Best-fit parameters are shown in Table 5. As in previous experiments, the PNIPA-20AA(5) nanohydrogel showed similar behaviour to PNIPA(5) but the collapse was less intense. Significant differences (*p*<0.05) were found between the *T_m_* of the PNIPA(5) and PNIPA-20AA(5) nanohydrogels, although changing water for a less polar solvent, such as methanol, prevented us from observing the *T_m_* shift due to the introduction of a hydrophilic monomer (AA) into the polymer.

**Table 5:** Parameters generated from fitting the Equation 24 to experimental data of optical density variation in PNIPA/AA nanohydrogel suspensions as a function of temperature (T, K) and the methanol volume fraction (MeOH, v/v).

	PNIPA(5)	PNIPA-20AA(5)
*T_m_* (K)	312.83±1.05^a^	306.48±1.67^b^
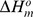 (KJ mol^−1^)	194.99±19.76^a^	183.32±26.04^a^
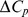 (KJ mol^−1^ K^−1^)	3.24±0.87^a^	5.07±1.50^a^
*m_1_*	240.94×10^−3^±27.70×10^−3^ a	312.68×10^−3^±49.37×10^−3^ a
*m_2_*	−51.99×10^−3^±4.32×10^−3^ a	−69.31×10^−3^±7.12×10^−3^ a
*m_3_*	−0.89×10^−3^±0.09×10^−3^ a	−0.67×10^−3^±0.16×10^−3^ a
r^2^	0.9862	0.9834

Values reported are the means±standard deviation (*n* = 2).

a,b Statistically significant differences (*p*<0.05) between nanohydrogels.

With respect to the effect of methanol, both pure PNIPA and PNIPA-co-AA nanohydrogels were more collapsed when the volume fraction of methanol (v/v) was about 0.40. When the methanol concentration was increased even more, the nanohydrogel re-swelled until it reached OD values similar to those obtained in pure water at low temperatures. However, the effect of temperature was different in the presence or absence of methanol (Figure 8). In the presence of a high concentration of methanol the increase in temperature had not effect on the polymer collapse and the particles remained swollen. This behaviour is related to the decrease in the dielectric constant due to the presence of the organic solvent. It can be hypothesized that methanol has a mask effect. Thus, the hydrophobic cores responsible for the nanohydrogel collapse could interact with the solvent, preventing the internal hydrophobic interactions in the polymer. This mask effect only happens when the dielectric constant of the medium is low enough at the highest methanol concentrations assayed.

The grey line in Figures 8A and 8C shows the variation in the critical methanol concentration over the temperature range assayed. The critical methanol fraction decreased with the temperature, and was 0.40 for PNIPA(5) and 0.35 for PNIPA-20AA(5) at 25°C, and decreased in both cases 0.08 units when the temperature was increased up to 37°C.

### Conclusions

The copolymerization of PNIPA with acrylic acid (AA) gives different structural properties to nanoparticles, such as increases in surface charge as well as the particle size and swelling capacity if the cross-linker is not present. Only when AA is present in the PNIPA nanohydrogel, are the swelling properties affected by the pH.

The presence of an organic solvent, such as methanol, modifies the behaviour of PNIPA particles in a non-linear way resulting in critical methanol concentration values that determine maximum collapse.

Modelling experimental results using a van't Hoff based equation is a suitable procedure for estimating some thermodynamic parameters consistently with the DSC measurements, and also allows the molecular weight of the polymer to be estimated. In addition, mathematical models obtained provided a suitable tool to understand which factors influence the release of active compounds from PNIPA nanohydrogels and then chose the right nanohydrogel composition as function of the physicochemical characteristics of the food product and storage conditions.
